# First Reported Prairie Dog–to-Human Tularemia Transmission, Texas, 2002

**DOI:** 10.3201/eid1003.030695

**Published:** 2004-03

**Authors:** Swati B. Avashia, Jeannine M. Petersen, Connie M. Lindley, Martin E. Schriefer, Kenneth L. Gage, Marty Cetron, Thomas A. DeMarcus, David K. Kim, Jan Buck, John A. Montenieri, Jennifer L. Lowell, Michael F. Antolin, Michael Y. Kosoy, Leon G. Carter, May C. Chu, Katherine A. Hendricks, David T. Dennis, Jacob L. Kool

**Affiliations:** * Centers for Disease Control and Prevention, Atlanta, Georgia, USA; † Texas Department of Health, Austin, Texas, USA; ‡ Centers for Disease Control and Prevention, Fort Collins, Colorado, USA; § Texas Department of Health, Arlington, Texas, USA; ¶ Colorado State University, Fort Collins, Colorado, USA

**Keywords:** tularemia, zoonoses, animals, occupational health, *Francisella tularensis*

## Abstract

A tularemia outbreak, caused by *Francisella tularensis* type B, occurred among wild-caught, commercially traded prairie dogs. *F. tularensis* microagglutination titers in one exposed person indicated recent infection. These findings represent the first evidence for prairie-dog-to-human tularemia transmission and demonstrate potential human health risks of the exotic pet trade.

Tularemia is a zoonosis affecting more than 150 wildlife species, including prairie dogs, squirrels, cats, and humans (*1*–*3*). Tularemia is caused by the bacterium *Francisella tularensis*, which exists in two main types. Type A is found almost exclusively in North America and is highly virulent in humans. Type B exists throughout North America, Asia, and Europe and is less virulent in humans (*4*). Tularemia vaccines have been used to protect military and laboratory personnel at high risk for exposure but are not available for the general population (*5*).

Humans can acquire tularemia through contact with infected animals (*2**,**3**,**6*). Although not previously documented, prairie dog–to-human-transmission is a concern because thousands of wild prairie dogs are captured annually in the United States and sold as exotic pets worldwide (*7*).

In mid-July 2002, a die-off began among wild-caught, black-tailed prairie dogs (*Cynomys ludovicianus*) (Figure 1) at a commercial exotic pet distributorship in Texas (facility A). On July 29, one of the dead prairie dogs tested positive for *F. tularensis* (*8*). Hundreds of potentially infected prairie dogs had already been distributed to other states and exported internationally. Epidemiologic and microbiologic investigations were initiated on August 1. We report on the epidemiologic findings; the microbiologic investigation is reported separately (*9*).

**Figure 1:**
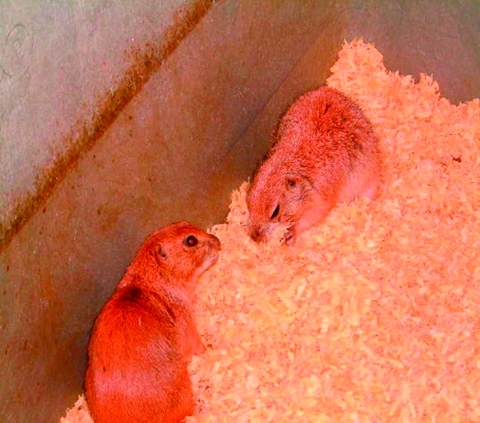
Black-tailed prairie dogs (*Cynomys ludovicianus*).

## The Study

### Animal Investigation

Facility A’s purchasing and shipping records were reviewed and the staff interviewed. All involved states and countries were notified of the outbreak, asked to identify the status of prairie dogs from the suspected shipments, and submit tissue samples for testing.

All prairie dogs at facility A, prairie dogs distributed within Texas from facility A since June 2002, and other dead and free-roaming exotic species at facility A were retrieved; live animals were euthanized, and all were tested for *F. tularensis* by direct fluorescence assay (DFA) and culture on cysteine heart agar with 9% chocolatized blood media (*9*). All recovered isolates were subtyped by using a polymerase chain reaction (PCR) assay (*9*).

Trappers who supplied prairie dogs to facility A in May and June 2002 were interviewed, and prairie dogs from their respective facilities in Texas and South Dakota were euthanized and tested for tularemia. South Dakota trapping sites suspected to be a potential source of the outbreak were also investigated.

Investigation of facility A on August 2 indicated a variety of exotic species crowded within a 2,500 square foot building. We found 163 remaining prairie dogs in four groups: sick and dying prairie dogs (bin 1), healthy-appearing prairie dogs (bin 2 and cages), prairie dog carcasses (frozen), and escaped prairie dogs roaming free around the facility. The bins were metal, uncovered, 2.5 feet tall and 5 feet in diameter, with 50–100 prairie dogs per bin. In addition, several other exotic animals were found roaming free or dead.

**Figure 2:**
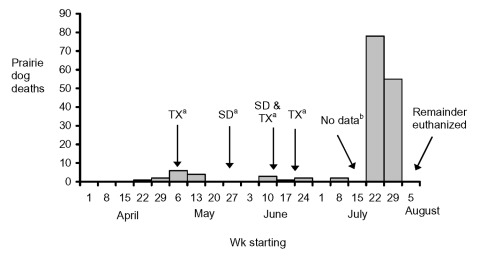
Weekly prairie-dog deaths at facility A, Texas, April–August, 2002. ^a^ Arrows represent prairie dog shipments arriving at facility A from Texas (TX) and South Dakota (SD).^b^ No data are available for the week of July 15, when the outbreak was first noticed by facility A staff.

According to shipping records, approximately 3,600 prairie dogs passed through facility A during January through July 2002. In July, an estimated 250 prairie dog deaths occurred compared with approximately 25 deaths over the previous 6 months (Figure 2). On August 1, shipments to and from facility A were halted.

Necropsies on all 163 prairie dogs remaining in facility A indicated clinical signs of oropharyngeal tularemia in all the dead and most of the euthanized sick animals, suggesting transmission through ingestion. Many of the dead animals had been cannibalized. *F. tularensis* was isolated from 61 animals (Table 1). Of these, 60 isolates came from prairie dogs remaining in facility A, including one prairie dog roaming free in the facility, and one isolate came from a privately owned prairie dog purchased from a Texas pet shop supplied by facility A. All of the isolates were identified as type B.

**Table 1:** Diagnostic results for all animals tested in association with tularemia outbreak in prairie dogs, Texas, 2002

Location	Species	No. animals tested	Confirmed positive^a^
Facility A	Prairie dogs	163	61
Retrieved from other Texas facilities	Prairie dogs	7	1
Czech Republic	Prairie dogs	6	1
Trapper facility, TX	Prairie dogs	8	0
Trapper facility, SD	Prairie dogs	2	0
Michigan	Prairie dogs	2	0
Facility A	Chinchilla, sugarglider, hedgehog, red squirrel, eastern chipmunk	16	0
Field investigation, Mellette County, SD	Prairie dogs, deer mice, white-footed mice, grasshopper mice, ground squirrel, jack rabbit, meadow vole	90	0

^a^Prairie dogs were confirmed positive on recovery of an isolate with characteristic growth on cysteine heart agar with 9% chocolatized blood and positive testing of the isolate by direct fluorescent antibody or polymerase chain reaction.

During June through July 2002, more than 1,000 prairie dogs were distributed from facility A to locations in 10 U.S. states and 7 other countries (Table 2). By early August, 100 prairie dogs, those shipped to the Czech Republic, remained unsold: of these, approximately 30 were dead on arrival, 30 were ill, and evidence of cannibalism had been noted within the shipment. All living animals were euthanized.

**Table 2:** Numbers of prairie dogs distributed from facility A to U.S. states and countries in Europe and Asia, June–July, 2002

Locations	No. prairie dogs
United States	
Texas	115
Illinois	26
Ohio	20
Washington	18
Arkansas	12
Nevada	12
West Virginia	12
Michigan	2
Florida	1
Mississippi	1
Europe	
the Netherlands	400
Belgium	250
Czech Republic	100
France	2
Portugal	1
Asia	
Japan	328
Thailand	2

Of the prairie dogs distributed from facility A to other U.S. states, specimens were received from two prairie dogs sent to Michigan; serum samples from both tested negative for tularemia (Table 1). The Netherlands and Belgium retrieved 4 and 10 prairie dogs, respectively, for serologic testing and culture of tissue samples; all were reported to be negative. The Czech Republic tested six prairie dogs for tularemia: one was positive by isolation of *F. tularensis* in culture, and five were presumptively positive by polymerase chain reaction (PCR). The Czech *F. tularensis* isolate was identified as type B, indistinguishable from the Texas isolates by restriction fragment length polymorphism analysis (*9*).

All healthy-appearing prairie dogs in bin 2 and cages, as well as other exotic animals roaming free or found dead in facility A tested negative for tularemia, demonstrating that outbreak propagation required direct contact with infected prairie dogs. Prairie dogs collected from Texas trappers, South Dakota trappers, and trapping sites all tested negative.

### Human Investigation

A human case was defined as a fourfold change in serial *F. tularensis* antibody titers from serum samples obtained at least 14 days apart, with at least one titer ≥1:128, in an exposed person. Paired serum samples were tested with an *F. tularensis* microagglutination assay. Anyone who transported, handled, bought, or cleaned the cages of prairie dogs from facility A since June 2002 was considered exposed. Exposed persons in Texas and other U.S. states were given a standardized questionnaire to assess infection risk factors and symptoms during the 2 weeks after their exposure. To enhance case finding, periodic follow-up was maintained with health authorities in involved U.S. states and foreign countries.

Twenty-two exposed persons were identified in Texas: 5 worked at facility A, 13 worked at other Texas facilities supplied by facility A, 3 worked at a veterinary care center and necropsied a prairie dog originating from facility A, and 1 privately owned an infected prairie dog originating from facility A. In interviews with 20 of 22 exposed persons, 6 (32%) reported recent prairie-dog bites, 7 (37%) ate or drank without handwashing after contact with prairie dogs, and 13 (67%) handled prairie dogs or cleaned cages barehanded. Although gloves and soap were available to employees, none of the involved Texas facilities had formal written policies enforcing proper handwashing, wearing gloves, or prohibiting eating or drinking in animal care areas.

During their exposure interval, 14 of 20 exposed persons interviewed reported having >2 nonspecific symptoms that can be consistent with tularemia: headache, sore throat, myalgias, stiff neck, fever, chills, cough, and swollen glands. Initial serologic testing on blood samples obtained 1 week to 2 months after initial exposure from 19 of 22 persons in Texas identified a positive *F. tularensis* titer of 1:128 in a 24-year-old man, who was an animal handler at facility A. All other persons tested negative, and no new positive titers were identified from follow-up samples obtained 1–2 months later from 9 of 19 persons. Except for the animal handler, other symptomatic persons had spontaneous resolution of symptoms or other diagnoses for their symptoms. The animal handler’s 1-month follow-up titer persisted at 1:128; however, a fourfold decline in titer, from 1:128 to 1:32, was documented for samples obtained 4 and 6 months after the initial titer, indicating recent exposure to *F. tularensis*. The animal handler had begun working at facility A in June 2002 and had handled dead and dying prairie dogs barehanded. He denied prior potential tularemia exposures, such as hunting, having tick bites, or owning a pet. Additionally, he denied having received a tularemia vaccine, which could have explained the elevated titer. During our investigation, the animal handler reported having an afebrile upper respiratory infection-like illness atypical of tularemia, with sore throat, cough productive of green sputum, and mild chest discomfort but no interruption of work or leisure activities. His symptoms began 12 days after the last prairie dog shipment arrived at facility A and 1 week before the die-off, and they resolved after oral fluoroquinolone therapy.

Health authorities in other states and countries reported no illness in exposed persons. Six months after the outbreak occurred, follow-up calls to health authorities in the involved U.S. states indicated no new human cases. No serologic testing was performed on exposed persons outside of Texas.

## Conclusions

Our investigation demonstrated the first evidence that prairie dogs can transmit tularemia to humans. The animal handler’s atypical symptoms and unclear route of infection might be because he was exposed to the less virulent subspecies type B. Studies have documented higher rates of *F. tularensis* seropositivity among animal trappers from tularemia-endemic areas, and many of the trappers were asymptomatic (*10*).

This outbreak highlights health risks to humans who handle wild-caught animals and underscores the speed with which exotic species and virulent pathogens can be transported worldwide (*11*). A number of public health risks associated with the exotic pet trade were observed at facility A. Prairie dogs were crowded in large bins, allowing unnaturally close contact and propagation of the outbreak through cannibalism. A variety of wild-caught and captive-bred exotic animals were also held in close quarters, providing opportunity for diseases to jump species. This risk for disease transmission between species was heightened because several exotic animals were able to roam free and comingle.

Until recently in the United States, no federal regulations existed to protect humans from the domestic distribution and sale of infected, wild-caught animals; a ban against transport and sale of prairie dogs and certain other rodent species was implemented on June 11, 2003, in response to a monkeypox outbreak in the Midwest (*12*). Many states forbid capture and sale of native wildlife species, including prairie dogs; however, states that do permit trapping and sale do not have regulations to address the human risk of acquiring zoonoses.

This incident and others, such as transmission to humans of plague from prairie dogs, monkeypox from prairie dogs, and salmonellosis from African pygmy hedgehogs, highlight the importance of developing strategies to reduce human risk from the domestic and international sale of infected, wild-caught animals (*13*–*16*). Strategies might include educating the public, standardizing exotic animal husbandry practices, restricting trade to animals bred in captivity, or banning sale of wild-caught animals. As a result of this investigation, Japan banned prairie dog importation as of March 2003. We recommend that U.S. states and other countries review and strengthen their regulations governing the transport and sale of prairie dogs and other exotic pets.

## References

[1] La Regina M, Lonigro J, Wallace M. *Francisella tularensis* infection in captive, wild caught prairie dogs. Lab Anim Sci. 1986;36:78–80. 3702338

[2] Magee JS, Steele RW, Kelly NR, Jacobs RF. Tularemia transmitted by a squirrel bite. Pediatr Infect Dis J. 1989;8:123–5. 2704604

[3] Quenzer RW, Mostow SR, Emerson JK. Cat-bite tularemia. JAMA. 1977;238:1845. 10.1001/jama.238.17.1845 578542

[4] Tärnvik A, Sandström G, Sjöstedt A. Epidemiological analysis of tularemia in Sweden 1931-1993. FEMS Immunol Med Microbiol. 1996;13:201–4. 10.1016/0928-8244(95)00104-2 8861029

[5] Dennis DT, Inglesby TV, Henderson DA, Bartlett JG, Ascher MS, Eitzen E, Tularemia as a biological weapon: medical and public health management. JAMA. 2001;285:2763–73. 10.1001/jama.285.21.2763 11386933

[6] Callaway GD, Peterson SS, Good JT. Tularemia in southwest Missouri: a report and discussion of seventy-eight cases. Mo Med. 1954;51:906–9. 13213885

[7] Roberts C. Prairie dogs said to bring pox on America. Washington Times. June 12, 2003. Available from: http://www.washingtontimes.com/national/20030611-115121-8325r.htm

[8] Centers for Disease Control and Prevention. Public health dispatch: outbreak of tularemia among commercially distributed prairie dogs, 2002. MMWR Morb Mortal Wkly Rep. 2002;51:688. 12233912

[9] Petersen JM, Schriefer ME, Carter LG, Zhou Y, Sealy T, Bawiec D, Laboratory analysis of tularemia in wild trapped, commercially traded prairie dogs, Texas, 2002. Emerg Infect Dis. 2004;▪▪▪:10. 15109407 10.3201/eid1003.030504 PMC3322795

[10] Levesque B, De Serres G, Higgins R, D’Halewyn MA, Artsob H, Grondin J, Seroepidemiologic study of three zoonoses (leptospirosis, Q fever, and tularemia) among trappers in Quebec, Canada. Clin Diagn Lab Immunol. 1995;2:496–8. 7583933 10.1128/cdli.2.4.496-498.1995 PMC170188

[11] Constantine DG. Geographic translocation of bats: known and potential problems. Emerg Infect Dis. 2003;9:17–21. 12533276 10.3201/eid1309.020104 PMC2873759

[12] HHS bans rodent imports from Africa; prohibits domestic commerce in rodents and prairie dogs [press release]. Washington, DC: Department of Health and Human Services; June 11, 2003. Available from: http://www.hhs.gov/news/press/2003pres/20030611a.html

[13] Craig C, Styliadis S, Woodward D, Werker D. African pygmy hedgehog—associated *Salmonella tilene* in Canada. Can Commun Dis Rep. 1997;23:129–31. 9376819

[14] Centers for Disease Control and Prevention. African pygmy hedgehog-associated salmonellosis—Washington, 1994. MMWR Morb Mortal Wkly Rep. 1995;44:462–3. 7776952

[15] Alexander JL. Plague, pet prairie dogs—USA (Texas). ProMed. July 10, 1998. Accessed at: http://www.promedmail.org, archive number: 19980710.1303.

[16] Centers for Disease Control and Prevention. Fatal human plague—Arizona and Colorado, 1996. MMWR Morb Mortal Wkly Rep. 1997;46:618–20. 9218646

